# Structural Insights into the Intrinsically Disordered GPCR C-Terminal Region, Major Actor in Arrestin-GPCR Interaction

**DOI:** 10.3390/biom12050617

**Published:** 2022-04-21

**Authors:** Myriam Guillien, Assia Mouhand, Aurélie Fournet, Amandine Gontier, Aleix Martí Navia, Tiago N. Cordeiro, Frédéric Allemand, Aurélien Thureau, Jean-Louis Banères, Pau Bernadó, Nathalie Sibille

**Affiliations:** 1 Centre de Biologie Structurale (CBS), University of Montpellier, CNRS, INSERM, 34090 Montpellier, France; guillien@cbs.cnrs.fr (M.G.); mouhand@cbs.cnrs.fr (A.M.); fournet@cbs.cnrs.fr (A.F.); gontier@dynamic-biosensors.com (A.G.); elloto13@gmail.com (A.M.N.); tiago.cordeiro@itqb.unl.pt (T.N.C.); allemand@cbs.cnrs.fr (F.A.); bernado@cbs.cnrs.fr (P.B.); 2 Instituto de Tecnologia Química e Biológica António Xavier (ITQB), Universidade NOVA de Lisboa, Av. da República, 2780-157 Oeiras, Portugal; 3 Beamline SWING, Synchrotron SOLEIL, L’Orme des Merisiers, Saint-Aubin BP 48, 91190 Gif-sur-Yvette, France; aurelien.thureau@synchrotron-soleil.fr; 4 IBMM, UMR5247 CNRS, Pôle Chimie Balard Recherche, 1919 route de Mende, CEDEX 5, 34293 Montpellier, France; jean-louis.baneres@umontpellier.fr

**Keywords:** GPCR, arrestin, intrinsically disordered proteins or regions (IDPs/IDRs), NMR

## Abstract

Arrestin-dependent pathways are a central component of G protein-coupled receptor (GPCRs) signaling. However, the molecular processes regulating arrestin binding are to be further illuminated, in particular with regard to the structural impact of GPCR C-terminal disordered regions. Here, we used an integrated biophysical strategy to describe the basal conformations of the C-terminal domains of three class A GPCRs, the vasopressin V2 receptor (V2R), the growth hormone secretagogue or ghrelin receptor type 1a (GHSR) and the β2-adernergic receptor (β2AR). By doing so, we revealed the presence of transient secondary structures in these regions that are potentially involved in the interaction with arrestin. These secondary structure elements differ from those described in the literature in interaction with arrestin. This suggests a mechanism where the secondary structure conformational preferences in the C-terminal regions of GPCRs could be a central feature for optimizing arrestins recognition.

## 1. Introduction

**Figure 1:**
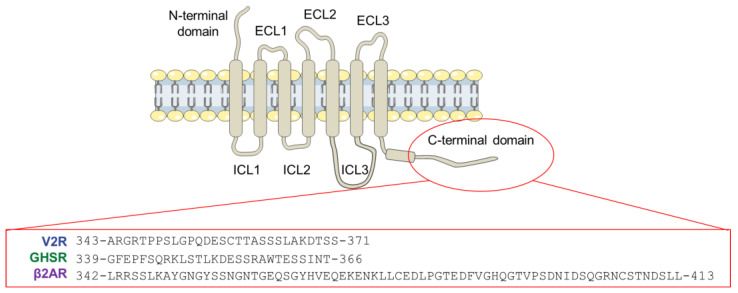
**Schematic representation of a GPCR.** GPCRs share a common core domain with seven transmembrane helices (7TM) linked by extracellular (ECL) and intracellular (ICL) loops. These loops, as well as the N- and C-terminal domains, are variable in length and sequence. The sequences of the three peptides used in this study are indicated just below.

G protein-coupled receptors (GPCRs) are integral membrane proteins involved in signal transduction. They are central in the cellular response for a wide range of extracellular ligands, such as hormones, nucleotides, lipids, ions, photons, and neurotransmitters [1]. Their signaling outcome regulates a large number of biological functions and, therefore, their dysfunctions are linked to various pathologies [2]. Consequently, GPCRs are the target of a third of the current clinical drugs [3]. Their functional diversity comes from the existence of a large number of GPCRs (~800 members in humans), classified according to their sequence and phylogenetic analyses [4]. While they share a highly conserved core domain (7TM) composed of seven transmembrane helices connected by three extracellular (ECL) and intracellular (ICL) loops, their extracellular N- and intracellular C-termini, as well as the loops, are highly variable in both sequence and length (Figure 1) [5,6].

Upon extracellular ligand binding, GPCR conformational rearrangements allow G protein association and G protein dependent signaling initiation. Besides G proteins, GPCRs can trigger other signaling pathways by interacting with arrestin, i.e., desensitization, internalization, and receptor trafficking [7]. Arrestin interaction requires GPCR C-terminal domain (GPCR-Cter) phosphorylation by specific G protein-coupled receptor kinases (GRKs). The current model of GPCR:arrestin interaction, the so-called phospho-barcode model [8,9], states that, upon activation, GPCRs become phosphorylated at specific sites on their C-terminal domain, and this impacts their interaction with arrestin, in turn affecting its conformation and, ultimately, the intracellular signal triggered by arrestin. However, we are just beginning to grasp the mechanistic basis of this mode of functioning as experimental demonstrations are still scarce even for the best-studied GPCRs.

GPCR:arrestin complex formation leads to arrestin activation through conformational changes (for a review see [10]). However, putative conformational changes of GPCR-Cter induced by GRK phosphorylation or arrestin binding are still poorly described. These C-terminal regions are predicted to behave as intrinsically disordered regions (IDRs) [11,12]. Intrinsically disordered proteins (IDPs) and IDRs are highly dynamic proteins/regions with a low content of transient secondary structures, which make their structural characterization difficult [13]. Atomic structures of GPCR-arrestin complex revealed that the C-terminus of the vasopressin 2 receptor (V2R) and rhodopsin are partially folded on arrestin surface [13,14,15,16]. This suggests a mechanism by which the IDR undergoes conformational changes upon post-translational modification and/or binding to its target [14]. Indeed, IDPs/IDRs most likely contain some pre-formed secondary structure elements that are often involved in the recognition of specific partners and modulate the affinity [15].

In order to better understand how the structural features of the GPCR C-terminal regions could impact on their functional role, we characterized the structure of the truncated C-terminal regions of three GPCRs, namely the vasopressin V2 receptor (V2R), the ghrelin receptor type 1a (GHSR) and the β2-adernergic receptor (β2AR) (Figure 1). These three class-A receptors are important therapeutic targets [2] and are representative of the two different classes of arrestin binders [16]. The first class, which includes β2AR and GHSR, forms a transient complex with arrestin that dissociates near the plasma membrane. Thus, arrestin does not internalize with the receptor. In contrast, the second class, which includes V2R, forms a more stable complex allowing the internalization of the whole assembly.

Here, we confirmed the disordered nature of these three GPCR-Cters and the presence of transient secondary structures by a set of biophysical tools: circular dichroism (CD), multi angle light scattering (MALS), and small angle X-ray scattering (SAXS). Then, we used nuclear magnetic resonance (NMR) to probe the conformational and dynamic preferences at residue level, using a set of complementary experiments, such as secondary chemical shifts (SCS), scalar (J) and residual dipolar couplings (RDCs), paramagnetic relaxation enhancement (PRE) and relaxation [17,18,19,20]. We show that the three C-terminal regions of the chosen GPCRs displayed different transient secondary structures, which could be involved in arrestin binding. These regions could act as short linear motifs (SLiMs), partner recognition IDP segments that are embedded in poorly conserved, disordered regions. By comparison with crystallographic structures of synthetic peptides in interaction with arrestin [21,22,23], our results suggest that structural changes in these putative SLiMs occur either after phosphorylation of the C-terminal region or upon arrestin binding. 

## 2. Materials and Methods

### 2.1. Sample Preparation

The (human) cDNA coding for the C-terminus of V2R (343–371), GHSR (339–366) and β2AR (342–413) were ordered to GeneArt^®^ gene synthesis (Life Technologies, Courtaboeuf, France). They were cloned into the expression vector pETM33 for GHSR-Cter and V2R-Cter, while β2AR-Cter was cloned into pET1a expression vector. Vectors were transfected into *E. Coli* BL21 DE3 strain (Fisher Scientific S.A.S., Illkirch, France) and (His)6-GST-tagged proteins were expressed in self-inducible medium N5052 or in minimal medium M9 by adding 2 mM IPTG when the OD600 reached 0.6. Labeling with 15N and/or 13C was carried out by growing cells in the previous media with 99% ^15^NH_4_Cl (CortecNet, Voisins-Le-Bretonneux, France) and/or 99% ^13^C_6_-Glucose (Cambridge Isotope Laboratories Inc., Tewksbury, MA, USA) as unique source of nitrogen and carbon, respectively. After harvesting, bacteria were resuspended and lysed by sonication in 20 mM Tris 20 pH 7.5, 300 mM NaCl and 2 mM dithiothreitol (DTT) (buffer A) supplemented with anti-protease (Complete^®^ EDTA free tablet (Sigma-Aldrich Chimie S.A.R.L.-Roche, Saint Quentin Fallavier, France)). Cell debris was removed by centrifugation and the soluble fraction was loaded onto 5 mL HisTrap FF column (Cytiva Europe GmbH, Velizy Villacoublay, France). The resin was washed with buffer A containing 10 mM imidazole and proteins were eluted with a linear gradient of buffer A supplemented with 500 mM imidazole. The combined eluate was dialyzed over night at 4 °C in 1 L of buffer A with the 3C PreScission protease (ratio protease:protein 1:100 (w/w)) or TEV protease (ratio protease:protein 1:50 (w/w)) for the pETM33 and pET1a constructs, respectively. Proteases and His-GST tags were removed using gravity column containing 2.5 mL Ni-sepharose (GE Healthcare). Untagged proteins were concentrated using a 2 kDa Millipore concentrator and injected into a HiLoad 16/60 Superdex 75 exclusion column (GE Healthcare) with SEC buffer (50 mM Bis-Tris pH 6.7, 50 mM NaCl, 2 mM DTT). The purified proteins were then concentrated to 200–300 μM using Millipore concentrator and aliquots were flash-frozen in 50 mM Bis-Tris pH 6.7, 50 mM NaCl, 1 mM EDTA (EthyleneDiamineTetraAcetic acid), 0.5 mM TCEP (Tris(2-CarboxyEthyl)Phosphine) and stored at −80 °C until use.

Concentration of V2R-Cter was determined by refractometry after gel filtration, while concentrations of β2AR-Cter and GHSR-Cter were estimated using absorbance at 280 nm.

### 2.2. Bioinformatic Analyses

#### 2.2.1. Disorder Prediction

The disorder state of the three C-termini was determined using the Charge-Hydropathy plot computed by PONDR [24]. Disordered propensity of amino-acid were predicted using PONDR-FIT [25], PrDOS [26], SPOT-Disorder [27,28], Espritz-NMR [29], DisPro [30], and DISOPRED3 [31] predictors.

#### 2.2.2. Secondary Structure Prediction

Secondary structure predictions were computed with different servers: SOPMA [32], PSIPRED [33], JPRED4 [34], PSSpred [35], SPOT1D [36], SPIDER3 [37].

### 2.3. Size Exclusion Chromatography-Multi-Angle Light Scattering (SEC-MALS)

The experiments were performed at 25 °C using a Superdex 75 10/300 GL column (GE HealthCare) connected to a miniDAWN-TREOS light scattering detector and an Optilab T-rEX differential refractive index detector (Wyatt Technology, Santa Barbara, CA, USA). The column was equilibrated in 50 mM BisTris pH 6.7, 50 mM NaCl, 1 mM TCEP and 0.5 mM EDTA buffer filtered at 0.1 µM, and the SEC-MALS system was calibrated with a sample of Bovine Serum Albumin (BSA) at 1 mg/mL. Samples at 1.5 mM, 0.6 mM, and 0.7 mM were prepared for V2R-Cter, GHSR-Cter, and β2AR-Cter, respectively. For each GPCR-Cter, 40 µL of sample were injected at 0.5 mL/min. Data acquisition and analyses were performed using the ASTRA software (Wyatt).

### 2.4. Circular Dichroism (CD)

Far UV-spectra of the C-termini were recorded in a quartz cuvette (path length 0.1 cm) at 0.08 mg/mL in H_2_O at 20 °C using a Chirascan. The ellipticity was scanned from 190 to 260 nm with an increment of 0.5 nm, an integration time of 3 s, and a constant band-pass of 1 nm. Data were treated using Chirascan and, after substraction of the buffer signal, were converted to mean residue ellipticity ([θ]_MRW_, mdeg.cm^2^.dmole^−1^) using Equation (1) [38]:

[θ]_MWR_ = [(θ × Mw)/(L × C × 10)]/(n − 1) (1)

where θ is the ellipticity (mdeg), Mw is the molecular weight (g/mol), L is the cell length (cm), C is the protein concentration (mg/mL), and n is the number of peptide bonds.

### 2.5. Small-Angle X-ray Scattering (SAXS) Measurement and Analysis

Synchrotron radiation SAXS data were acquired for GPCR-Cters at the SWING beamline at the SOLEIL synchrotron (Saint-Aubin, France) [39] using an X-ray wavelength of 1.03 Å and a sample-to-detector distance of 1.99 m. Samples were measured at 15 °C and at two concentrations, 5 mg/mL and 10 mg/mL, for all GPCR-Cters, in 50 mM BisTris pH 6.7, 50 mM NaCl and 2 mM DTT buffer. Before exposure to X-rays, 45 µL of sample were injected into 3 mL Superdex 75 5/150 GL column (GE HealthCare) at 0.2 mL/min, pre-equilibrated into the same buffer as the samples. The intensity was measured as function of the magnitude of the scattering vector, *s*, using Equation (2) [40]:

*s* = 4π.sin(θ)/λ, (2)

where θ is the scattering angle and λ is the X-ray wavelength.

The scattering patterns of the buffer were recorded before the void volume of the column (1 mL). The scattering profiles measured covered a momentum transfer range of 0.002 < *s* < 0.5 Å^−1^. Data were processed using CHROMIX from ATSAS [41] software package to automatically select frames corresponding to buffer and sample, and performed buffer subtraction. The scaled and averaged SAXS curves were analyzed using Primus from ATSAS software package.

### 2.6. Nuclear Magnetic Resonance (NMR) Spectroscopy

All NMR experiments were performed on a Bruker Bruker Avance III 700 MHz spectrometer, except for the 3D assignment of β2AR-Cter performed on a 800 MHz, and for the ^3^J_HNHA_ of GHSR-Cter performed on a 500 MHz. The 700 MHz and 800 MHz spectrometers are equipped with a cryogenic triple-resonance (^1^H, ^15^N, ^13^C) probe and shielded z-gradients. All NMR experiments were recorded at 20 °C in a buffer (named NMR buffer) composed of 50 mM Bis-Tris pH 6.7, 150 mM NaCl, 1 mM EDTA, 0.5 mM TCEP, 5% D_2_O (Eurisotop), and 5 mM DSS-d6 (2,2-dimethyl-2-silapentane-5-sulfonate, Sigma) as internal reference [42]. All experiments used the pulse sequences provided by Bruker Topspin 3.2. Squared cosine apodization was used in indirect dimensions, prior to zero-filling and Fourier transformation using TOPSPIN (version 4.0.6, Bruker) and data processing was performed using NMRFAM-SPARKY (version 1.414, [43]). For each NMR experiments, concentrations of GPCR-Cters were indicated in Table S1. For all NMR experiments, data were measured for all residues of C-terminus regions excepted proline residues, the residue A339 of β2AR-Cter, and the first N-terminal residue.

#### 2.6.1. Backbone Assignment

For the sequential assignment of the ^13^C/^15^N GPCR C-terminus of V2R, GHSR and β2AR, HNCO, HN(CA)CO, HNCA, HN(CO)CA, CBCA(CO)NH and HNCACB triple resonance 3D experiments were recorded. H_N_, N, CO, C_α_ and C_β_ nuclei of all residues were assigned, expected the first N-terminal residue, A339 for β2AR-Cter and proline residues.

#### 2.6.2. Secondary Chemical Shift (SCS)

^13^C_α_ and ^13^C_β_ chemical shifts were used to calculate Secondary Chemical Shift (SCS) by subtraction of experimental chemical shifts (from the 3D experiment) from random-coil chemical shift computed by POTENCI database [44,45]. SCS were calculated for all residues of C-terminal domains excepted proline residues, the N-terminal residue, A339 for β2AR-Cter, and the last residue.

#### 2.6.3. ^3^J_HNHA_ Coupling

^3^J_HNHA_ scalar coupling measurements were obtained according to Vuister and Bax [46]. Briefly, HNHA experiments were recorded on ^15^N-labelled GPCR C-termini. Intensity of the cross-peak (S_cross_) and intensity of the corresponding diagonal peak (S_diag_) were extracted using Sparky. They were used to calculate the ^3^J_HNHA_ scalar coupling of each amino acid using the Equation (3):

(S_cross_/S_diag_) = −tan^2^(2π × ^3^J_HNHA_ × ξ) (3)

where 2ξ is the total evolution time for the homonuclear ^3^J_HNHA_ coupling, which has been set to 26.1 ms.

^3^J_HNHA_ scalar coupling were measured for all residues of C-terminal tails excepted proline residues and the first glycine residue. Random coil scalar coupling were predicted using RC_3JHNHa server [47].

#### 2.6.4. ^1^H-^15^N Residual Dipolar Couplings (RDCs)

RDCs were obtained by recording 2D IPAP HSQC spectra [48] in isotropic and anisotropic media. The anisotropic media were obtained by adding a 5% (*w*/*v*) mixture of polyoxyethylene 5-lauryl ether (PEG/C12E5) (Sigma) and 1-hexanol (Sigma) in a molar ratio of 0.85 [49] or by adding ~20 mg/mL of filamentous phage Pf1 (Asla biotech) [50]. Spectra were recorded on ^15^N-labelled GPCR C-termini in alcohol and phage media. ^1^D_NH_ dipolar couplings were measured from the difference of doublet peak positions in the ^15^N dimension measured in the anisotropic (J + D) and isotropic (J) spectra. 

#### 2.6.5. ^15^N Relaxation Experiments

Relaxation data were measured on ^15^N-labelled GPCR C-termini for all residues except proline residues and the two first N-terminal residues. Heteronuclear ^15^N{^1^H}-NOE values were determined from two experiments with on- (saturated spectrum) and off-resonance ^1^H saturation (unsaturated spectrum) that were recorded in an interleaved manner. The saturation time by 120° pulses (~10 kHz) was set to 6 s and the recycle delay to 6 s. NOEs values were obtained from the ratio of intensities measured in the saturated (I) and unsaturated (I_0_) spectra. Longitudinal (R_1_) and transversal (R_2_) relaxation rates were measured through acquisition of ^15^N-HSQC spectra with different relaxation delays: 10, 50, 100, 200, 400, 600, 800, 1000 ms for R_1_, and 16, 32, 64, 96, 160, 240, 480, 640 ms for R_2_. For each peak, the intensity was fitted to a single exponential decay using Sparky [43] to obtain the relaxation parameters. For all relaxation parameters, three residues at N- and C-termini were discarded from the calculation of average values due to their inherent higher flexibility.

#### 2.6.6. Paramagnetic Relaxation Enhancement (PRE)

The C378A or C406A variants of ^15^N β2AR-Cter were labeled on the remaining cysteine using 3-(2-Iodoacetamodi)-proxyl (Merck). Paramagnetic samples were recorded with a recycling delay of 2 s. Reference diamagnetic samples were recorded in the same conditions after the addition of 5 mM fresh ascorbic acid, pH 6.7, in the NMR tube. PRE were analyzed by measuring the peak intensity ratios (I_para_/I_dia_) between two ^15^N-HSQC spectra of paramagnetic and diamagnetic samples. The theoretical profile expected for a strictly random coil polymer was calculated according to [51].

#### 2.6.7. Ensemble Calculations

Ensembles of explicit models were generated using Flexible-Meccano (FM) [52], which sequentially builds peptide planes based on amino acid specific conformational propensity and a simple volume exclusion term. To account for deviations from a random-coil description, different structure ensembles of 50,000 conformers were computed including user-defined local conformational propensities in different regions of the protein. Local conformational propensities were first localized using the consensus of all NMR data, and then were adjusted by comparing back-calculated and experimental ^1^D_HN_ RDCs to get the lowest X^2^ (for more details [53]). For β2AR-Cter, a long-range contact of 15 Å between two regions affected by the probe, i.e., from residues 338 to 357 and from residues 367 to 386 (regions in grey in Figure S7b), was introduced to get a better agreement between back-calculated and experimental ^1^D_HN_ RDCs.

## 3. Results

### 3.1. The Disordered C-Termini of V2R, GHSR, and β2AR Contain Transient Secondary Structures

**Figure 2:**
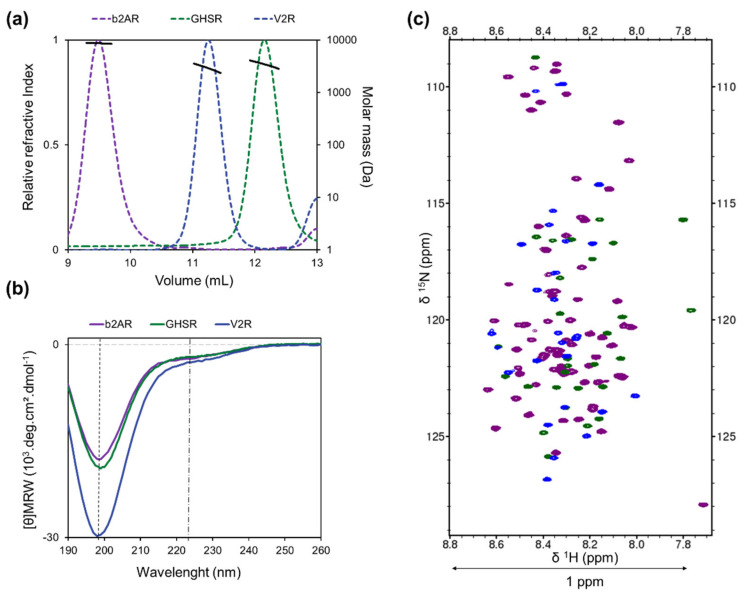
**The C-terminal domain of V2R-Cter (blue), GHSR-Cter (green), and β2AR-Cter (purple) are monomeric and disordered**. The color code is maintained for all panels. (**a**) SEC-MALS curves (colored dashed lines). Molar masses derived from MALS are indicated by thick black line (right axis); (**b**) far-UV circular dichroism (CD) spectra (colored lines) present a minimum around 200 nm (black dashed line) and a shoulder at 220 nm (black dashed and dotted line) characteristic of disordered proteins with transient secondary structure content; (**c**) ^15^N-HSQC spectra display a low proton spectral dispersion (~1 ppm) typical of IDPs. HSQCs were recorded on 300 µM samples at 700 MHz and 20 °C, in 50 mM Bis-Tris pH 6.7, 150 mM NaCl buffer.

**Figure 3:**
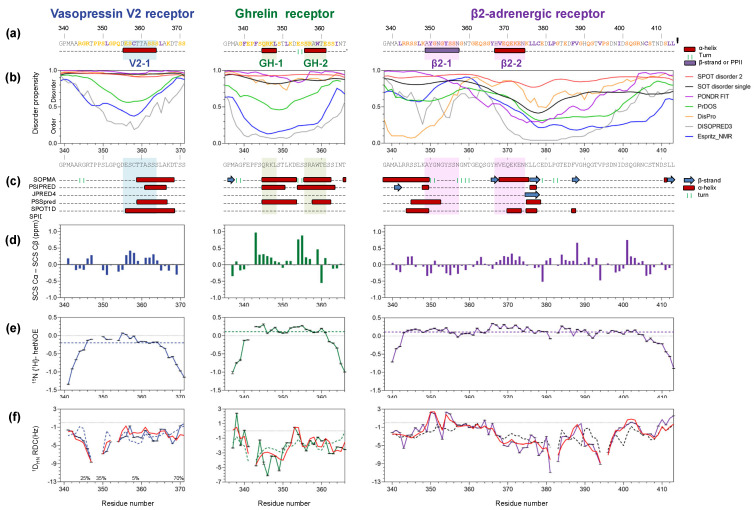
**Bioinformatics predictions, secondary structure propensity, backbone dynamics, and RDC conformational profile of the disordered V2R-Cter (blue), GHSR-Cter (green), and β2AR-Cter (purple)**. (**a**) GPCR C-termini are composed of more than 50% disorder-promoting residues (in orange). Ordered-promoting residues are indicated in purple. Secondary structures obtained by the consensus of all NMR data are indicated under the sequence and highlighted according to their respective color code. Helix, β-strand, extended conformation (β-strand or polyproline helix 2, PPII), and turns are represented as red cylinder, blue arrow, purple box, and green bars, respectively; (**b**) disorder prediction by SPOT-Disorder 2 (red), SPOT-Disorder Single (black), PONDR-FIT (purple), PrDOS (green), DisPro (orange), DISOPRED 3 (grey), and Espritz-NMR (blue). The disorder/order threshold (0.5) is indicated in black line; (**c**) secondary structure prediction by SOPMA, PSIPRED, JPRED4, PSSpred, SPOT 1D, and SPIDER 3 web-servers are represented for helices (red cylinder), strands (blue arrow), and turns (green); (**d**) computed secondary structure SCS Cα-SCS Cβ using random coil chemical shifts from POTENCI; (**e**) heteronuclear ^15^N{^1^H}-NOEs are represented with colored curves and the average with dash colored lines; (**f**) back-calculated and experimental ^1^D_HN_ RDCs. Comparison of experimental RDC (colored according to the GPCR-Cter color code) and back-calculated values computed with FM on a random-coil ensemble (dash color line) and biased ensemble (red line) and populated as indicated in the figure for helical structure (highlighted in red), extended structure (highlighted in blue), or turn (highlighted in green). Spectra were recorded at 700 MHz and 20 °C, in 50 mM Bis-Tris pH 6.7, 150 mM NaCl buffer.

As is the case for many C-terminal domains of GPCRs [54], the C-terminal regions of V2R, GHSR, and β2AR are shown to be disordered (Figure 2) and are predicted to contain transient secondary structures by various computational tools (Figure 3). In fact, they are composed of more than 50% of disorder-promoting residues (Arg, Gly, Gln, Ser, Pro, Glu, and Lys) (Figure 3a) [55]. In the Charge-Hydropathy plot [56] (Figure S1), V2R-Cter is at the disorder-order boundary, while β2AR-Cter (pink) and GHSR-Cter (green) appeared in the cluster of disordered proteins (yellow). Sequence based disorder prediction by a set of six predictors (Table S2) showed an overall disordered state (values higher than 0.5) for the three C-termini (Figure 3b). More specifically, prediction by PrDOS, DISOPRED3, and Espritz-NMR showed less disordered regions from residues ~351 to 365 for V2R-Cter and from residues ~343 to 359 for GHSR-Cter (Figure 3b). β2AR-Cter is also predicted to be less disordered from ~345 to 356 and from ~369 to 405 according to PrDOS, DISOPRED3, and Espritz-NMR predictors (Figure 3b). Interestingly, these regions are predicted to contain helical conformations by a set of distinct secondary structure predictors (Figure 3c, Table S3).

In order to characterize these domains experimentally, we expressed and purified the intrinsically disordered and soluble C-terminal regions of V2R (343–371), GHSR (339–355), and β2AR (342–413). SEC-MALS analysis revealed a single elution peak for each protein at a volume that corresponded to the volume of standard proteins with a molecular mass greater than 13 kDa. However, the masses derived from a MALS analysis are 3.9 (±3.2%), 3.4 (±3.7%), and 8.6 (±1.1%) kDa for V2R-Cter, GHSR-Cter, and β2AR-Cter, respectively (Figure 2a). This is in agreement with the expected molecular weight of their monomeric forms (3.3, 3.6, and 8.2 kDa, respectively). Molecular masses were confirmed by mass spectrometry (MS) with 3.264, 3.672, and 8.175 kDa obtained for V2R-Cter, GHSR-Cter, and β2AR-Cter, respectively. The behavior of V2R-Cter, GHSR-Cter, and β2AR-Cter on a SEC column is typical of a disordered protein, with a smaller elution volume than expected for globular proteins of the same molecular mass [57]. In addition, far UV CD spectra showed a minimum around 198 nm, characteristic of unfolded proteins [58] (Figure 2b), and a negative shoulder around 222 nm, suggesting the presence of residual secondary structures [59] (Figure 2b). The Kratky plots extracted from SAXS data were typical of disordered regions with no clear maximum and a monotonic increase along the momentum transfer range (Figure S2) [18,60,61]. Additionally, ^15^N-HSQC spectra of the three C-terminal regions showed a reduced amide proton spectral dispersion (around 1 ppm) typical of a disordered protein (Figure 2c) [18,19].

Altogether, bioinformatics analyses and experimental data confirm the disordered nature of the C-terminal regions of V2R, GHSR, and β2AR [62]. Furthermore, these results suggest the presence of residual secondary structures. 

### 3.2. Location of the Transient Secondary Structures in V2R, GHSR, and β2AR C-Termini

In order to localize the transient secondary structures of these three GPCR C-terminal regions, we performed a NMR study, as described in [53]. Before NMR investigation, the backbone assignments were performed on the studied C-terminal domains of GPCRs (Figure S3) (BMRB accession codes, respectively: 51318, 51317, and 51316). Then, we used a consensus of four NMR parameters to highlight the secondary structure content. First, ^13^C secondary chemical shifts (SCS), which are highly sensitive to the backbone conformations, were computed (Figure 3d and Figure S4b–g) [45,63]. Three-bond H_N_-H_a_ J-coupling constants (^3^J_HNHA_), which are related to the φ angles of the polypeptide chain, were compared to random coil scalar coupling [46,47]. Residual dipolar couplings (RDCs), which are related to the orientation of the amide backbone vector to the magnetic field [64], give information on the location and secondary structure type. These data were also compared to back-calculated RDCs on random-coil ensembles computed with Flexible-Meccano [52,65]. Finally, dynamic parameters, such as heteronuclear ^15^N{^1^H}-NOEs, longitudinal (R_1_), and transverse (R_2_) relaxation rates, give information on local backbone mobility on the ps–ns timescale, while R_2_ is also sensitive to motions on the µs to ms timescale (chemical or conformational exchange processes). Thus, R_2_/R_1_ ratio reveals slow conformational motions or conformational/chemical exchange. Depending on the NMR experiments, the GPCR-Cter sample concentrations varied, but did not affect the structure (Table S1, Figure S3).

#### 3.2.1. Vasopressin V2 Receptor C-Terminal Domain (V2R-Cter)

For V2R-Cter, a central helical region was identified from residues 356 to 364, called V2-1 from now on (Figure 3). Indeed, ^13^C SCS presented positive values in this region (Figure 3d). The presence of this helix is in agreement with disorder and secondary structure predictions (Figure 3b,c). Globally, in V2R-Cter, 32% of the residues displayed ^3^J_HNHA_ scalar coupling values below 6 Hz, normally assigned to helical conformations, and the rest were between 6 and 8 Hz, consistent with a random coil (RC) polypeptide chain (Figure S4i). Moreover, experimental ^3^J_HNHA_ scalar couplings for the V2-1 region were lower than the predicted ones (Figure S4h) [47], substantiating its helical conformation. Further evidence for transient secondary structure elements in V2-1 came from dynamical parameters. V2R-Cter showed low heteronuclear ^15^N{^1^H}-NOE values as expected for a disordered polypeptide. However, in the V2-1 region, heteronuclear NOE values were above the average (−0.09 ± 0.01), suggesting an enhanced rigidity in the region (Figure 3e). Residues of V2-1 adopted larger R_2_ values than the average (2.80 ± 0.05 Hz), suggesting the presence of slow dynamic processes. R_1_ and R_2_/R_1_ also displayed slightly higher values for V2-1 than their averages, 1.75 ± 0.03 Hz and 1.59 ± 0.04, respectively (Figure S5). This transient helical secondary structure in V2-1 was also highlighted by ^1^D_NH_ RDC values. RDCs were measured in alcohol mixture (C_12_E_5_/hexanol media) and in filamentous bacteriophage Pf1. However, the homogeneity of the aligned sample was checked on the quadrupolar splitting of D_2_O and was higher in alcohol medium (27 Hz) than in Pf1 medium. While RDC values were mainly negative, as observed in disordered proteins, the segment encompassing V2-1 displayed higher values than those expected for a random coil, which was in agreement with the presence of a helix in this region (Figure 3f). 

#### 3.2.2. Ghrelin Receptor C-Terminal Domain (GHSR-Cter)

For GHSR-Cter we identified two transient secondary structures: from residues 345 to 348 (called GH-1) and from residues 356 to 361 (called GH-2) (Figure 3). These regions were predicted to form helices (Figure 3c) and SCS showed positive values, suggesting helical conformations (Figure 3d). The SCS profile also presented high positive values for 354-355 that could be related to the presence of a turn as predicted by the secondary structure predictor SOPMA. ^3^J_HNHA_ scalar couplings confirmed that GHSR-Cter was mostly disordered (50%) with transient helical conformations (46%) and a small portion of extended conformation (4%) (Figure S4i). The comparison of experimental and random coil ^3^J_HNHA_ scalar couplings was in agreement with the presence of transient helices in GH-1 and GH-2 (Figure S4h). Additionally, in these two regions, heteronuclear NOE, R_1_, R_2_, and R_2_/R_1_ showed higher values than their averages, 0.11 ± 0.01; 2.11 ± 0.04 Hz; 3.17 ± 0.06 Hz and 1.49 ± 0.04, respectively (Figure 3e and Figure S5). This indicated less flexibility in these regions, suggesting some local structuration. When measuring RDCs, a small interaction between the alcohol mixture and GH-1 region (chemical shift differences > 0.01 ppm between ^15^N-HSQC spectra) was observed (Figure S6); thus we analyzed RDCs measured in phage Pf1 medium (quadrupolar splitting of 18 Hz). ^1^D_NH_ RDCs exhibited lower (GH-1 region) and higher (GH-2 region) values than those expected from a random coil, suggesting an extended and a helical conformation in these regions, respectively (Figure 3f). 

#### 3.2.3. β2-Adrenergic Receptor C-Terminal Domain (β2AR-Cter)

For β2AR-Cter, we identified two regions forming secondary structures: from residues 349 to 357 (called β2-1) and from 368 to 376 (called β2-2). They were predicted to be more ordered and to contain helical secondary structures (Figure 3a,b). However, SCS presented negative values for β2-1 and positive values for β2-2, corresponding to an extended and a helical conformation, respectively (Figure 3d). ^3^J_HNHA_ scalar couplings were consistent with an overall unstructured protein (73% of the residues) with a small content of helical (25%) and extended conformations (1%) (Figure S4i). In β2-2, experimental scalar couplings were lower than those predicted for a random coil, suggesting a helical conformation in this region. Note that glycines are not predicted by the RC_3JHNHa server; thus scalar couplings in β2-1 could not be properly compared to random-coil values [47] (Figure S4h). In β2-1 and β2-2, heteronuclear NOE and R_2_ values were above their average, 0.11 ± 0.01 and 3.44 ± 0.01 Hz, respectively. This indicated restricted flexibility in these regions (Figure 2e and Figure S6). However, in β2-2, R_1_ remained flat. Consequently, R_2_/R_1_ displayed larger values than the average (1.83 ± 0.03), suggesting conformational fluctuations on the µs–ms time-scale. RDC measurement in Pf1 medium showed a small interaction with β2-2 (chemical shift differences > 0.01 ppm) (Figure S6); thus we used RDC data extracted from the alcohol mixture (quadrupolar splitting of 31 Hz). Compared to random coil values computed with FM, experimental RDCs showed more positive values in β2-1 and more negative values in β2-2 (Figure 3f), suggesting a helical and an extended conformation, respectively. However, RDCs are not only sensitive to local structuration, such as SCS, but they can also probe long-range transient contacts [66,67]. Indeed, paramagnetic relaxation enhancement (PRE) data of the C406A variant, where the paramagnetic probe in C378 lies just after β2-2 region, showed reduction of the intensity ratio in the N-terminal part of β2AR-Cter, including β2-1 region and its N-flanking region, revealing long-range contacts between the N-terminal part of β2AR-Cter and β2-2 C-flanking region (respectively, from residues 338 to 357 and 367 to 386). PRE affected regions are highlighted in grey in Figure S7b. These long-range interactions could affect the overall profile of RDCs, probably explaining the absence of consensus between SCS and RDC data (see below). The second PRE dataset, measured in the C378A variant, where the paramagnetic probe lies at the C-terminal region (C406), did not induce substantial reduction of the intensity ratio in β2AR-Cter, indicating the overall disorder of the protein. Interestingly, a slight reduction of intensity in the N-terminal part β2AR-Cter confirmed the presence of fuzzy long-range contacts between the N- and C-terminal parts of the protein (Figure S7c). 

#### 3.2.4. Conformational Ensemble of GPCR-Cters

To further illuminate the presence of transient secondary structures or turns in the three C-terminal domains of GPCRs, we built biased ensembles using Flexible Meccano (FM) (Figure 3f). Ensembles of 50,000 conformers were built for V2R-Cter, GHSR1a-Cter, and β2AR-Cter, respectively, and were used to compute back-calculated RDCs (Figure 3f). In these ensembles, we added as constraints the transient secondary structures determined by the consensus analyses of all NMR data to improve the agreement between experimental and back-calculated RDCs. Then, the population of the local conformational propensities and turns were adjusted by monitoring the agreement using χ^2^. The quality of these ensembles was evaluated by comparing the back-calculated RDCs with the experimental ones and was optimized until obtaining the lowest possible χ^2^ (near 1). RDCs were measured in alcohol mixture for V2R-Cter and β2AR-Cter or in phage Pf1 medium for GHSR1a-Cter. For V2R-Cter, the best agreement (χ^2^ = 1.41) was obtained when 5% of α-helix was imposed in V2-1 and when poly-proline helices II (PPII) were added at 25%, 35%, and 70% in position 344–347, 349–350, and 368–370, respectively. For GHSR1a-Cter, the best ensemble (χ^2^ = 1.49) was obtained by adding 8% of α-helix in GH-2 and 20% of type 1 β-turn that was predicted for 353 to 354. Additional secondary structures were added (residues 337–338: 50% of type I β-turns, 340–341: 15% of type I β-turns: 343–344: 5% γ-turn; 363–366: 10% of β-strand). For β2AR-Cter, the lowest χ^2^ (χ^2^ = 1.36) was obtained when the long-range contact (15 Å) identified with PRE between regions surrounding β2-1 (from residues 338 to 357) and β2-2 (from residues 367 to 386) was incorporated to the model (see details in Materials and Methods 2.6.7., and PRE affected regions are highlighted in grey in Figure S7b). These results substantiate the presence of transient long-range interactions in β2AR-Cter. In β2-1, two type I β-turns at 50% were added, and in β2-2, a type II Poly-Proline helix (PPII) at 10% was imposed. This ensemble was also constrained by two other PPII in position 380–383 and 391–394, a type I β-turn in position 388–389 and a helix from residues 399 to 402.

In all C-terminal domains, the introduction of these secondary structures resulted in a better description of the experimental data, suggesting a more accurate description of the conformational ensembles.

## 4. Discussion and Conclusions

All GPCRs share a conserved and folded 7TM domain involved in the signal transmission. Conversely, the extracellular regions (N-terminal and C-terminal domains and loops) are rather diversified in length and sequence [4] and are involved in the functional properties of GPCRs, such as, respectively, ligand and partner binding. It is interesting to note that theses extracellular regions are predicted to contain intrinsically disordered regions (IDRs) [5,6], which could play key roles in GPCR interaction (for review [68,69]). Indeed, IDRs explore an astronomical number of conformations in solution that we assume in fast equilibrium, and very often contain pre-formed secondary structure elements. In the majority of cases, these transient secondary structures, or short linear motifs (SLIMs), are involved in the binding process with their partner [15], which makes IDPs very well suited agents for signaling processes, such as arrestin:GPCR interaction. Thus, the characterization of the structural features of these extracellular regions of GPCRs is crucial to reveal the molecular basis of signaling and cell regulation [70]. For instance, it was reported that the truncated C-terminal domain of GPR50 (GPR50-Cter) translocates to the nucleus and directly regulates gene transcription; thus the cleavage of this Cter has an unconventional signaling mode of GPCRs [70]. Intriguingly, this GPR50 receptor has been deeply remodeled through evolution by the mutation of numerous residues and by the addition of a long C-terminal domain. Indeed, the GPR50 homolog found in lower vertebrates was lacking the Cter of the human GPR50 [71]. This illustrates how the study of the isolated C-terminal domain of GPCRs is of relevant importance per se for understanding the multitude of signaling pathways regulated by the C-terminal domains of GPCR.

With the hypothesis that the C-terminal domains of GPCRs contain partially structured elements involved in signaling pathways, we characterized the free state of the C-terminal domains from three commonly studied GPCRs, the vasopressin V2 receptor (V2R-Cter), the ghrelin receptor type 1a (GHSR-Cter) and the β2-adernergic receptor (β2AR-Cter). These three class-A receptors are important therapeutic targets [2] and present different affinities for arrestins [16].

**Figure 4:**
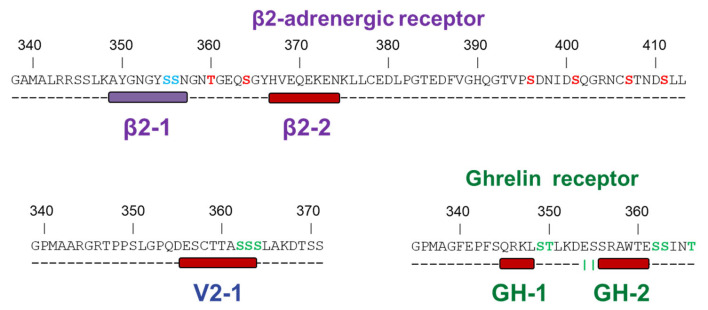
**The C-terminal regions of β2AR-Cter (purple), GHSR-Cter (green), and V2R-Cter (blue) revealed different transient secondary structures, which could be involved in arrestin binding**. GRK2 (red) and GRK6 (blue) phosphorylated sites according to [9] for β2AR, [72] for GHSR, and [73] for V2R are indicated in the sequence. Secondary structures obtained by NMR secondary structure consensus are indicated under the sequence, according to Figure 3.

The structural characterization of these disordered C-termini was challenging, due to their inherent flexibility, and required the synergistic application of several biophysical tools. Firstly, by SAXS, MALS, and CD, we confirmed that V2R-Cter, GHSR-Cter, and β2AR-Cter are IDRs containing secondary structures. Then, we used NMR in combination with computationally generated ensembles, to locate and identify these residual secondary structure elements. The C-terminus of V2R displayed a central helix from residues 356 to 364 (V2-1). The C-terminus of GHSR encompassed two helixes from 345 to 348 (GH-1) and from residues 356 to 361 (GH-2). The C-terminus of β2AR contained two structured regions: an extended conformation from 349 to 357 residues (β2-1) and a helix from 368 to 376 (β2-2) (Figure 4). Moreover, the propensities of these secondary structures were low (~25% on average), which illustrate the high flexibility of GPCR-Cters and their ability to adopt distinct conformations in solution, a general feature of IDPs/IDRs.

V2R-Cter, GHSR-Cter, and β2AR-Cter are variable in length and in sequence (Figure 1). However, it is interesting to note that their residual secondary structures either encompass or are next to residues known to be phosphorylated by GRKs (Figure 4). It is accepted that the phosphates of the C-terminal domains of GPCRs are important for the interaction with arrestin (for a review see [10]). This strongly suggests that the transient secondary structures that we have identified are directly involved in arrestin binding. 

**Figure 5:**
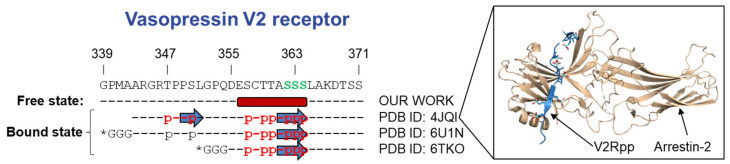
**A conformational change must occur in the C-terminal region of V2R (blue) upon binding to arrestin and/or phosphorylation**. Comparison of the free, in solution state of V2R-Cter to its bound state identified in complexes between a fully phosphorylated phospho-peptide of vasopressin V2 C-terminus (V2Rpp) and arrestin-2. On top is represented the sequence of human V2R C-terminus. Residues known to be phosphorylated by GRKs are colored in green [73]. Below, V2R-Cter sequences used in each work are represented in dashed lines. Residues encompassing the binding regions are identified in red and phosphorylated residues are noted as p. Stars (*) indicate that the V2R C-terminus is fused to another receptor (PDB: 6U1N, muscarinic receptor; 6TKO: β1-adrenergic receptor). Helices and β-strands are represented as red cylinders and blue arrows, respectively. In the frame, the PDB structure of V2R:arrestin-2 complexes is shown [21]. Phosphorylated residues of V2Rpp (in blue) are highlighted as sticks.

Interestingly, V2-1 region has been characterized in complex with arrestin in crystallographic and cryo-EM structures (Figure 5) [21,22,74]. These structures were obtained using a fully synthetic phospho-peptide of V2R C-terminal domain (V2Rpp), truncated or attached to other receptors (chimeric receptor) (Figure 5), and each of these complexes was stabilized with a Fab30 antibody. In these structures, the central region of V2Rpp adopts a β-strand that interacts with the N-domain of arrestin. Here, we show that in solution, this SLiM displays a helical structure instead of an extended conformation. This result suggests that a conformational change must occur upon binding to arrestin and/or phosphorylation, a feature that has been found in several SLiMs [75]. Moreover, this structural transition might be at the basis of the molecular regulation of GPCR:arrestin interaction. Indeed, β-strand formation has been proposed to serve as a general mechanism by which arrestins recognize the phosphorylated carboxy-terminal domains of receptors [21]. In the pre-structuration profile of GHSR-Cter and β2AR-Cter, only GH-2 and β2-2 are in helical conformations as found for the V2-1 region. Thus, we can hypothesize that these regions interact with arrestin with a similar mechanism to the one found for V2R, and that their basal conformation changes upon arrestin binding and/or phosphorylation. 

Until now, the free states of GPCR C-termini were poorly characterized due to their high flexibility. Comparison of our data with GPCRs:arrestin complexes showed that a conformational change is expected after GRK phosphorylation and/or arrestin binding. The phospho-barcode model states that distinct GRK phosphorylation patterns at the C-termini of GPCRs lead to distinct arrestin conformations and outcome functions [8,9,72,76]. Thus, we can anticipate that phosphorylation would dictate the signaling of GPCRs by modulating the folding of their C-termini and/or their folding upon binding. Phosphorylation has already been described as a regulator of IDP folding mechanism for biological function [77]. To test this hypothesis, the characterization of the secondary structure profile for each phosphorylation pattern of GPCR C-termini and the comparison with the basal profile described in this study will be the key to understanding how arrestin dependent signaling pathways are modulated. 

## Data Availability

The ^1^H, ^15^N and ^13^C assignment of β2AR, GHSR and V2R C-termini are available in the BioMagResBank (http://www.bmrb.wisc.edu) under the respective BMRB accession codes 51316, 51317 and 51318 (deposited on 9 February 2022).

## Supplementary Materials

The following are available online at https://www.mdpi.com/article/10.3390/biom12050617/s1. Table S1: Concentration used for the NMR characterization of GPCR C-termini of V2R-Cter (blue), GHSR-Cter (green), and β2AR-Cter (purple); Table S2: Related to Figure 3, Comparison of the disorder predictors used in this study; Table S3: Related to Figure 3, Comparison of the secondary structure predictors used in this study. Figure S1: Charge-Hydropathy plot; Figure S2: Related to Figure 2, SAXS data of V2R-Cter (blue, left), GHSR-Cter (green, middle) and β2AR-Cter (purple, right); Figure S3: Related to Figure 3, Assignments of V2R-Cter (blue, left), GHSR-Cter (green, middle) and β2AR-Cter (purple, right); Figure S4: Related to Figure 3, Secondary chemical shift (SCS) and ^3^JHNHA scalar coupling measured for for V2R-Cter (blue, left), GHSR-Cter (green, middle) and β2AR-Cter (purple, right); Figure S5: Related to Figure 3e, Relaxation data of V2R-Cter (blue, left), GHSR-Cter (green, middle) and β2AR-Cter (purple, right); Figure S6: Related to Figure 3f, ^1^D_NH_ RDCs values of V2R-Cter (blue, left), GHSR-Cter (green, middle) and β2AR-Cter (purple, right); Figure S7: Related to Figure 3, PRE data of β2AR-Cter variants. Reference: [18,56,61,78].

## References

[1] Bockaert J., Philippe Pin J. (1999). Molecular Tinkering of G Protein–Coupled Receptors: An Evolutionary Success. EMBO J..

[2] Schöneberg T., Schulz A., Biebermann H., Hermsdorf T., Römpler H., Sangkuhl K. (2004). Mutant G-Protein-Coupled Receptors as a Cause of Human Diseases. Pharmacol. Ther..

[3] Hauser A.S., Attwood M.M., Rask-Andersen M., Schiöth H.B., Gloriam D.E. (2017). Trends in GPCR Drug Discovery: New Agents, Targets and Indications. Nat. Rev. Drug Discov..

[4] Fredriksson R. (2003). The G-Protein-Coupled Receptors in the Human Genome Form Five Main Families. Phylogenetic Analysis, Paralogon Groups, and Fingerprints. Mol. Pharmacol..

[5] Lagerström M.C., Schiöth H.B. (2008). Structural Diversity of G Protein-Coupled Receptors and Significance for Drug Discovery. Nat. Rev. Drug Discov..

[6] Fonin A.V., Darling A.L., Kuznetsova I.M., Turoverov K.K., Uversky V.N. (2019). Multi-Functionality of Proteins Involved in GPCR and G Protein Signaling: Making Sense of Structure-Function Continuum with Intrinsic Disorder-Based Proteoforms. Cell Mol. Life Sci..

[7] Gurevich V.V., Gurevich E.V. (2019). GPCR Signaling Regulation: The Role of GRKs and Arrestins. Front. Pharmacol..

[8] Ren X.-R., Reiter E., Ahn S., Kim J., Chen W., Lefkowitz R.J. (2005). Different G Protein-Coupled Receptor Kinases Govern G Protein and Beta-Arrestin-Mediated Signaling of V2 Vasopressin Receptor. Proc. Natl. Acad. Sci. USA.

[9] Nobles K.N., Xiao K., Ahn S., Shukla A.K., Lam C.M., Rajagopal S., Strachan R.T., Huang T.-Y., Bressler E.A., Hara M.R. (2011). Distinct Phosphorylation Sites on the 2-Adrenergic Receptor Establish a Barcode That Encodes Differential Functions of -Arrestin. Sci. Signal..

[10] Gurevich V.V., Gurevich E.V. (2019). The Structural Basis of the Arrestin Binding to GPCRs. Mol. Cell. Endocrinol..

[11] Venkatakrishnan A., Flock T., Prado D.E., Oates M.E., Gough J., Madan Babu M. (2014). Structured and Disordered Facets of the GPCR Fold. Curr. Opin. Struct. Biol..

[12] Jaakola V.-P., Prilusky J., Sussman J.L., Goldman A. (2005). G Protein-Coupled Receptors Show Unusual Patterns of Intrinsic Unfolding. Protein Eng. Des. Sel..

[13] Uversky V.N. (2002). What Does It Mean to Be Natively Unfolded?. Eur. J. Biochem..

[14] Wright P.E., Dyson H.J. (2015). Intrinsically Disordered Proteins in Cellular Signalling and Regulation. Nat. Rev. Mol. Cell Biol..

[15] Fuxreiter M., Simon I., Friedrich P., Tompa P. (2004). Preformed Structural Elements Feature in Partner Recognition by Intrinsically Unstructured Proteins. J. Mol. Biol..

[16] Oakley R.H., Laporte S.A., Holt J.A., Caron M.G., Barak L.S. (2000). Differential Affinities of Visual Arrestin, ΒArrestin1, and ΒArrestin2 for G Protein-Coupled Receptors Delineate Two Major Classes of Receptors. J. Biol. Chem..

[17] Dyson H.J., Wright P.E. (2004). Unfolded Proteins and Protein Folding Studied by NMR. Chem. Rev..

[18] Sibille N., Bernadó P. (2012). Structural Characterization of Intrinsically Disordered Proteins by the Combined Use of NMR and SAXS. Biochem. Soc. Trans..

[19] Delaforge E., Cordeiro T.N., Bernadó P., Sibille N., Webb G.A. (2018). Conformational Characterization of Intrinsically Disordered Proteins and Its Biological Significance. Modern Magnetic Resonance.

[20] Milles S., Salvi N., Blackledge M., Jensen M.R. (2018). Characterization of Intrinsically Disordered Proteins and Their Dynamic Complexes: From in Vitro to Cell-like Environments. Prog. Nucl. Magn. Reson. Spectrosc..

[21] Shukla A.K., Manglik A., Kruse A.C., Xiao K., Reis R.I., Tseng W.-C., Staus D.P., Hilger D., Uysal S., Huang L.-Y. (2013). Structure of Active β-Arrestin1 Bound to a G Protein-Coupled Receptor Phosphopeptide. Nature.

[22] Staus D.P., Hu H., Robertson M.J., Kleinhenz A.L.W., Wingler L.M., Capel W.D., Latorraca N.R., Lefkowitz R.J., Skiniotis G. (2020). Structure of the M2 Muscarinic Receptor–β-Arrestin Complex in a Lipid Nanodisc. Nature.

[23] Nguyen A.H., Thomsen A.R.B., Cahill T.J., Huang R., Huang L.-Y., Marcink T., Clarke O.B., Heissel S., Masoudi A., Ben-Hail D. (2019). Structure of an Endosomal Signaling GPCR–G Protein–β-Arrestin Megacomplex. Nat. Struct. Mol. Biol..

[24] Romero P., Obradovic Z., Li X., Garner E.C., Brown C.J., Dunker A.K. (2001). Sequence Complexity of Disordered Protein. Proteins.

[25] Xue B., Dunbrack R.L., Williams R.W., Dunker A.K., Uversky V.N. (2010). PONDR-FIT: A Meta-Predictor of Intrinsically Disordered Amino Acids. Biochim. Biophys. Acta (BBA) Proteins Proteom..

[26] Ishida T., Kinoshita K. (2007). PrDOS: Prediction of Disordered Protein Regions from Amino Acid Sequence. Nucleic Acids Res..

[27] Hanson J., Paliwal K., Zhou Y. (2018). Accurate Single-Sequence Prediction of Protein Intrinsic Disorder by an Ensemble of Deep Recurrent and Convolutional Architectures. J. Chem. Inf. Model..

[28] Hanson K., Paliwal K., Litfin T., Zhou Y. SPOT-Disorder2: Improved Protein Intrinsic Disorder Prediction by Ensembled Deep Learning—ScienceDirect. https://www.sciencedirect.com/science/article/pii/S1672022920300243.

[29] Walsh I., Martin A.J.M., Di Domenico T., Tosatto S.C.E. (2012). ESpritz: Accurate and Fast Prediction of Protein Disorder. Bioinformatics.

[30] Cheng J., Sweredoski M.J., Baldi P. (2005). Accurate Prediction of Protein Disordered Regions by Mining Protein Structure Data. Data Min. Knowl. Disc..

[31] Jones D.T., Cozzetto D. (2015). DISOPRED3: Precise Disordered Region Predictions with Annotated Protein-Binding Activity. Bioinformatics.

[32] Geourjon C., Deléage G. (1995). SOPMA: Significant Improvements in Protein Secondary Structure Prediction by Consensus Prediction from Multiple Alignments. Bioinformatics.

[33] Buchan D.W.A., Jones D.T. (2019). The PSIPRED Protein Analysis Workbench: 20 Years On. Nucleic Acids Res..

[34] Drozdetskiy A., Cole C., Procter J., Barton G.J. (2015). JPred4: A Protein Secondary Structure Prediction Server. Nucleic Acids Res..

[35] Yan R., Xu D., Yang J., Walker S., Zhang Y. (2013). A Comparative Assessment and Analysis of 20 Representative Sequence Alignment Methods for Protein Structure Prediction. Sci. Rep..

[36] Hanson J., Paliwal K., Litfin T., Yang Y., Zhou Y. (2019). Improving Prediction of Protein Secondary Structure, Backbone Angles, Solvent Accessibility and Contact Numbers by Using Predicted Contact Maps and an Ensemble of Recurrent and Residual Convolutional Neural Networks. Bioinformatics.

[37] Heffernan R., Yang Y., Paliwal K., Zhou Y. (2017). Capturing Non-Local Interactions by Long Short-Term Memory Bidirectional Recurrent Neural Networks for Improving Prediction of Protein Secondary Structure, Backbone Angles, Contact Numbers and Solvent Accessibility. Bioinformatics.

[38] Chemes L.B., Alonso L.G., Noval M.G., de Prat-Gay G., Uversky V.N., Dunker A.K. (2012). Circular Dichroism Techniques for the Analysis of Intrinsically Disordered Proteins and Domains. Intrinsically Disordered Protein Analysis: Volume 1, Methods and Experimental Tools.

[39] Thureau A., Roblin P., Pérez J. (2021). BioSAXS on the SWING Beamline at Synchrotron SOLEIL. J. Appl. Crystallogr..

[40] Blanchet C.E., Svergun D.I. (2013). Small-Angle X-ray Scattering on Biological Macromolecules and Nanocomposites in Solution. Annu. Rev. Phys. Chem..

[41] Manalastas-Cantos K., Konarev P.V., Hajizadeh N.R., Kikhney A.G., Petoukhov M.V., Molodenskiy D.S., Panjkovich A., Mertens H.D.T., Gruzinov A., Borges C. (2021). ATSAS 3.0: Expanded Functionality and New Tools for Small-Angle Scattering Data Analysis. J. Appl. Crystallogr..

[42] Wishart D.S., Sykes B.D. (1994). [12] Chemical Shifts as a Tool for Structure Determination. Methods in Enzymology.

[43] Lee W., Tonelli M., Markley J.L. (2015). NMRFAM-SPARKY: Enhanced Software for Biomolecular NMR Spectroscopy. Bioinformatics.

[44] Tamiola K., Acar B., Mulder F.A.A. (2010). Sequence-Specific Random Coil Chemical Shifts of Intrinsically Disordered Proteins. J. Am. Chem. Soc..

[45] Nielsen J.T., Mulder F.A.A. (2018). POTENCI: Prediction of Temperature, Neighbor and PH-Corrected Chemical Shifts for Intrinsically Disordered Proteins. J. Biomol. NMR.

[46] Vuister G.W., Bax A. (1993). Quantitative J Correlation: A New Approach for Measuring Homonuclear Three-Bond J(HNH.Alpha.) Coupling Constants in 15N-Enriched Proteins. J. Am. Chem. Soc..

[47] Shen Y., Roche J., Grishaev A., Bax A. (2018). Prediction of Nearest Neighbor Effects on Backbone Torsion Angles and NMR Scalar Coupling Constants in Disordered Proteins. Protein Sci..

[48] Ottiger M., Delaglio F., Bax A. (1998). Measurement OfJand Dipolar Couplings from Simplified Two-Dimensional NMR Spectra. J. Magn. Reson..

[49] Rückert M., Otting G. (2000). Alignment of Biological Macromolecules in Novel Nonionic Liquid Crystalline Media for NMR Experiments. J. Am. Chem. Soc..

[50] Hansen M.R., Mueller L., Pardi A. (1998). Tunable Alignment of Macromolecules by Filamentous Phage Yields Dipolar Coupling Interactions. Nat. Struct. Biol..

[51] Teilum K., Kragelund B.B., Poulsen F.M. (2002). Transient Structure Formation in Unfolded Acyl-Coenzyme A-Binding Protein Observed by Site-Directed Spin Labelling. J. Mol. Biol..

[52] Ozenne V., Bauer F., Salmon L., Huang J.R., Jensen M.R., Segard S., Bernado P., Charavay C., Blackledge M. (2012). *Flexible-meccano*: A Tool for the Generation of Explicit Ensemble Descriptions of Intrinsically Disordered Proteins and Their Associated Experimental Observables. Bioinformatics.

[53] Senicourt L., Le Maire A., Allemand F., Carvalho J.E., Guee L., Germain P., Schubert M., Bernadó P., Bourguet W., Sibille N. (2021). Structural Insights into the Interaction of the Intrinsically Disordered Co-Activator TIF2 with Retinoic Acid Receptor Heterodimer (RXR/RAR). J. Mol. Biol..

[54] Tovo-Rodrigues L., Roux A., Hutz M.H., Rohde L.A., Woods A.S. (2014). Functional Characterization of G-Protein-Coupled Receptors: A Bioinformatics Approach. Neuroscience.

[55] Dunker A.K., Lawson J.D., Brown C.J., Williams R.M., Romero P., Oh J.S., Oldfield C.J., Campen A.M., Ratliff C.M., Hipps K.W. (2001). Intrinsically Disordered Protein. J. Mol. Graph. Model..

[56] Uversky V.N., Gillespie J.R., Fink A.L. (2000). Why Are “Natively Unfolded” Proteins Unstructured under Physiologic Conditions?. Proteins.

[57] Uversky V.N., Uversky V.N., Dunker A.K. (2012). Size-Exclusion Chromatography in Structural Analysis of Intrinsically Disordered Proteins. Intrinsically Disordered Protein Analysis: Volume 2, Methods and Experimental Tools.

[58] Woody R.W. (2010). Circular Dichroism of Intrinsically Disordered Proteins. Instrumental Analysis of Intrinsically Disordered Proteins.

[59] Woody R.W., Fasman G.D. (1996). Theory of Circular Dichroism of Proteins. Circular Dichroism and the Conformational Analysis of Biomolecules.

[60] Bernadó P., Svergun D.I. (2011). Structural Analysis of Intrinsically Disordered Proteins by Small-Angle X-Ray Scattering. Mol. BioSyst..

[61] Cordeiro T.N., Herranz-Trillo F., Urbanek A., Estaña A., Cortés J., Sibille N., Bernadó P. (2017). Small-Angle Scattering Studies of Intrinsically Disordered Proteins and Their Complexes. Curr. Opin. Struct. Biol..

[62] Shimada I., Ueda T., Kofuku Y., Eddy M.T., Wüthrich K. (2019). GPCR Drug Discovery: Integrating Solution NMR Data with Crystal and Cryo-EM Structures. Nat. Rev. Drug Discov..

[63] Wishart D.S., Sykes B.D. (1994). The 13C Chemical-Shift Index: A Simple Method for the Identification of Protein Secondary Structure Using 13C Chemical-Shift Data. J. Biomol. NMR.

[64] Bax A., Kontaxis G., Tjandra N. (2001). Dipolar Couplings in Macromolecular Structure Determination. Methods in Enzymology.

[65] Bernadó P., Blanchard L., Timmins P., Marion D., Ruigrok R.W.H., Blackledge M. (2005). A Structural Model for Unfolded Proteins from Residual Dipolar Couplings and Small-Angle X-ray Scattering. Proc. Natl. Acad. Sci. USA.

[66] Bernadó P., Bertoncini C.W., Griesinger C., Zweckstetter M., Blackledge M. (2005). Defining Long-Range Order and Local Disorder in Native α-Synuclein Using Residual Dipolar Couplings. J. Am. Chem. Soc..

[67] Salmon L., Nodet G., Ozenne V., Yin G., Jensen M.R., Zweckstetter M., Blackledge M. (2010). NMR Characterization of Long-Range Order in Intrinsically Disordered Proteins. J. Am. Chem. Soc..

[68] Guillien M., le Maire A., Mouhand A., Bernadó P., Bourguet W., Banères J.-L., Sibille N. (2020). IDPs and Their Complexes in GPCR and Nuclear Receptor Signaling. Progress in Molecular Biology and Translational Science.

[69] Appadurai R., Uversky V.N., Srivastava A. (2019). The Structural and Functional Diversity of Intrinsically Disordered Regions in Transmembrane Proteins. J. Membr. Biol..

[70] Ahmad R., Lahuna O., Sidibe A., Daulat A., Zhang Q., Luka M., Guillaume J.-L., Gallet S., Guillonneau F., Hamroune J. (2020). GPR50-Ctail Cleavage and Nuclear Translocation: A New Signal Transduction Mode for G Protein-Coupled Receptors. Cell. Mol. Life Sci..

[71] Dufourny L., Levasseur A., Migaud M., Callebaut I., Pontarotti P., Malpaux B., Monget P. (2008). GPR50 Is the Mammalian Ortholog of Mel1c: Evidence of Rapid Evolution in Mammals. BMC Evol. Biol..

[72] Bouzo-Lorenzo M., Santo-Zas I., Lodeiro M., Nogueiras R., Casanueva F.F., Castro M., Pazos Y., Tobin A.B., Butcher A.J., Camiña J.P. (2016). Distinct Phosphorylation Sites on the Ghrelin Receptor, GHSR1a, Establish a Code That Determines the Functions of ß-Arrestins. Sci. Rep..

[73] Nobles K.N., Guan Z., Xiao K., Oas T.G., Lefkowitz R.J. (2007). The Active Conformation of Beta-Arrestin1: Direct Evidence for the Phosphate Sensor in the N-Domain and Conformational Differences in the Active States of Beta-Arrestins1 and -2. J. Biol. Chem..

[74] Lee Y., Warne T., Nehmé R., Pandey S., Dwivedi-Agnihotri H., Chaturvedi M., Edwards P.C., García-Nafría J., Leslie A.G.W., Shukla A.K. (2020). Molecular Basis of β-Arrestin Coupling to Formoterol-Bound β 1-Adrenoceptor. Nature.

[75] Tompa P., Schad E., Tantos A., Kalmar L. (2015). Intrinsically Disordered Proteins: Emerging Interaction Specialists. Curr. Opin. Struct. Biol..

[76] Mayer D., Damberger F.F., Samarasimhareddy M., Feldmueller M., Vuckovic Z., Flock T., Bauer B., Mutt E., Zosel F., Allain F.H.T. (2019). Distinct G Protein-Coupled Receptor Phosphorylation Motifs Modulate Arrestin Affinity and Activation and Global Conformation. Nat. Commun..

[77] Bah A., Vernon R.M., Siddiqui Z., Krzeminski M., Muhandiram R., Zhao C., Sonenberg N., Kay L.E., Forman-Kay J.D. (2015). Folding of an Intrinsically Disordered Protein by Phosphorylation as a Regulatory Switch. Nature.

[78] Cordeiro T.N., Herranz-Trillo F., Urbanek A., Estaña A., Cortés J., Sibille N., Bernadó P. (2017). Structural Characterization of Highly Flexible Proteins by Small-Angle Scattering. Adv. Exp. Med. Biol..

