# Supplementary material for: Structural Insights into the Intrinsically Disordered GPCR C-Terminal Region, Major Actor in Arrestin-GPCR Interaction
Source: Biomolecules. 2022 Apr 21;12(5):617. doi: 10.3390/biom12050617 (PMC9138321; doi:10.3390/biom12050617)
Supplement: Supplementary file 1 [file biomolecules-12-00617-s001.zip › biomolecules-1614788-supplementary.pdf]

# Structural insights into the intrinsically disordered GPCR C-terminal region, major actor in arrestin-GPCR interaction

**Myriam Guillien [1], Assia Mouhand [1], Aurélie Fournet [1], Amandine Gontier [1], Aleix Martí Navia [1], Tiago N. Cordeiro [1,2], Frédéric Allemand [1], Aurélien Thureau [3], Jean-Louis Banères [3], Pau Bernadó [1], and Nathalie Sibille [1,*]**

[1] Centre de Biologie Structurale (CBS), Univ Montpellier, CNRS, INSERM, Montpellier, France
[2] Instituto de Tecnologia Química e Biológica António Xavier (ITQB), Universidade NOVA de Lisboa, Av. da República, 2780-157 Oeiras, Portugal.
[3] Beamline SWING, Synchrotron SOLEIL, L'Orme des Merisiers, Saint-Aubin BP 48, 91190 Gif-sur-Yvette, France
[4] IBMM, UMR5247 CNRS, Pôle Chimie Balard Recherche, 1919 route de Mende, 34293 Montpellier cedex 5, France
* Correspondence: nathalie.sibille@cbs.cnrs.fr; Tel.: +33 4 67 41 77 05, Fax: +33 4 67 41 79 13

| Protein | 3D | $^3J_{HNHA}$ | relaxation | RDC alcohol | RDC Pf1 | PRE |
|---|---|---|---|---|---|---|
| V2R | 170 | 170 | 373 | 310 | 139 | NT |
| GHSR | 312 | 314 | 124 | 390 | 285 | NT |
| β2AR | 255 | 487 | 487 | 487 | 220 | 50 |

**Table S1: Concentration used for the NMR characterization of GPCR C-termini of V2R-Cter (blue), GHSR-Cter (green) and β2AR-Cter (purple)**. Concentration used in each experiment is indicated in µM. The letters NT mean that these experiments have not been carried out.

| Disordered predictor | Attributes | | Meta-predictor | Webserver |
|---|---|---|---|---|
| | Sequence alignment | Structural information | | |
| Pr Dos | Psi-Blast | No | Yes | http://prdos.hgc.jp/cgi-bin/top.cgi |
| PONDR FIT | No | No | Yes | http://original.disprot.org/metapredictor.php |
| DISOPRED3 | Psi-Blast | Psi-Pred | Yes | http://bioinf.cs.ucl.ac.uk/psipred/ |
| SPOT-disorder 2 | Psi-Blast + Hhblits | SPOT-1D | No | https://sparks-lab.org/server/spot-disorder2/ |
| SPOT-disorder single | No | No | No | https://sparks-lab.org/server/spot-disorder-single/ |
| Espritz NMR | No | No | No | http://old.protein.bio.unipd.it/espritz/ |
| DISpro | UniqueProt | Sspro + ACCpro | No | http://scratch.proteomics.ics.uci.edu/ |

**Table S2: Related to Figure 3. Comparison of the disorder predictors used in this study**. For each predictor (first column), disordered is predicted based on amino-acid sequence and can be completed with sequence alignment and/or secondary structure information computed with tools listed in the second and third column. Some of these servers use multiple predictors (meta-predictor) as indicated in the fourth column. Webservers are listed in the last column of the table.

| Predictor | Sequence alignment | Predicted secondary structure | Webserver |
|---|---|---|---|
| SOPMA | BLAST + Clustal | Helix, Strand, Turn, Coil | https://npsa-prabi.ibcp.fr/cgi-bin/npsa_automat.pl?page=/NPSA/npsa_sopma.html |
| PSIPRED | PSI-BLAST | Helix, Strand, Coil | http://bioinf.cs.ucl.ac.uk/psipred/ |
| JPRED4 | PSI-BLAST + HMMER | Helix, Strand, Coil | https://www.compbio.dundee.ac.uk/jpred/ |
| PSSpred | PSI-BLAST | Helix, Strand, Coil | https://zhanggroup.org/PSSpred/ |
| SPOT1D | PSI-BLAST + HHblists | Helix, Strand, Coil | https://sparks-lab.org/server/spot-1d/ |
| SPIDER3 | PSI-BLAST + HHblists | Helix, Strand, Coil | https://sparks-lab.org/server/spider3/ |

**Table S3: Related to Figure 3. Comparison of the secondary structure predictors used in this study**. Predictors (first column) use sequence alignments computed with tools listed in the second column, predicted secondary structures are listed in the third column. Webservers are reported in the last column of the table.

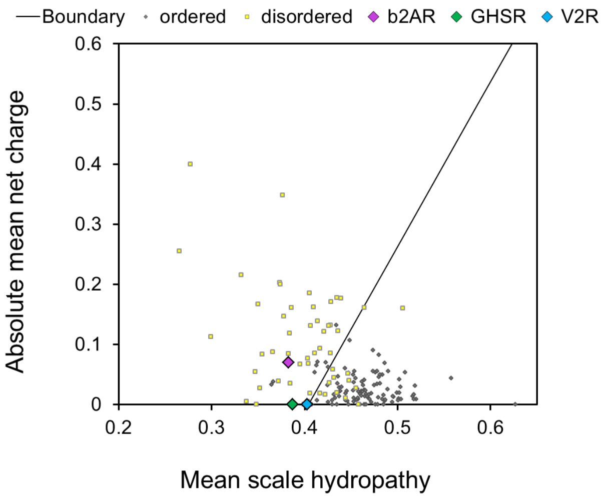


**Figure S1: Charge-Hydropathy plot [1]**. 54 disordered proteins are shown in yellow circles while 105 ordered proteins are shown in grey circles. The boundary between order/disorder (dark line) was calculated with the equation: $\overline{Hydropathy} = (\overline{net\ charge} + 1{,}151)/2{,}785$. V2R-Cter (blue), GHSR-Cter (green) and β2AR-Cter (pink) are represented with diamonds. This graph was generated using PONDR (www.pondr.com).

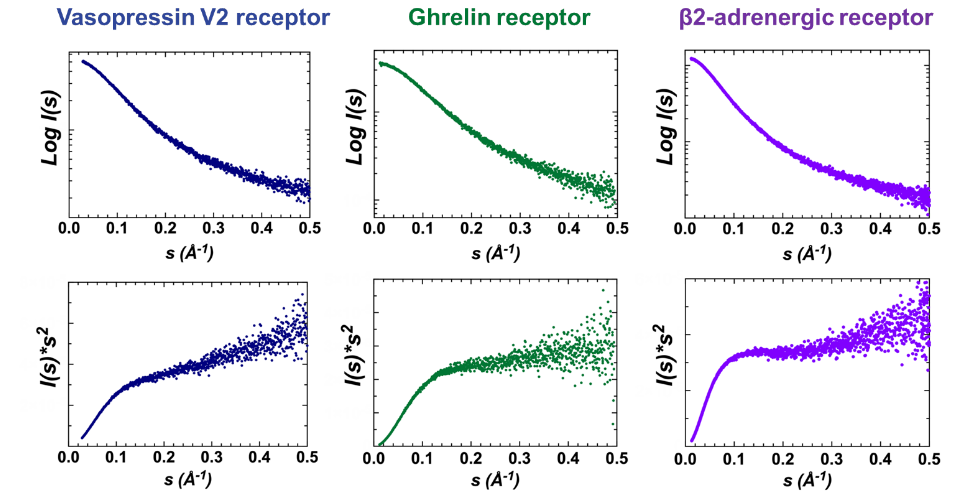


**Figure S2: Related to Figure 2. SAXS data of V2R-Cter (blue, left), GHSR-Cter (green, middle) and β2AR-Cter (purple, right).** Semi-logarithmic representation of the experimental SAXS curves versus scattering angle (upper panel) and Kratky plots (bottom panel). The Kratky plots of GPCR-Cters display a typical profile of IDP with no clear maximum and a monotone increase along the momentum transfer range [2–4].

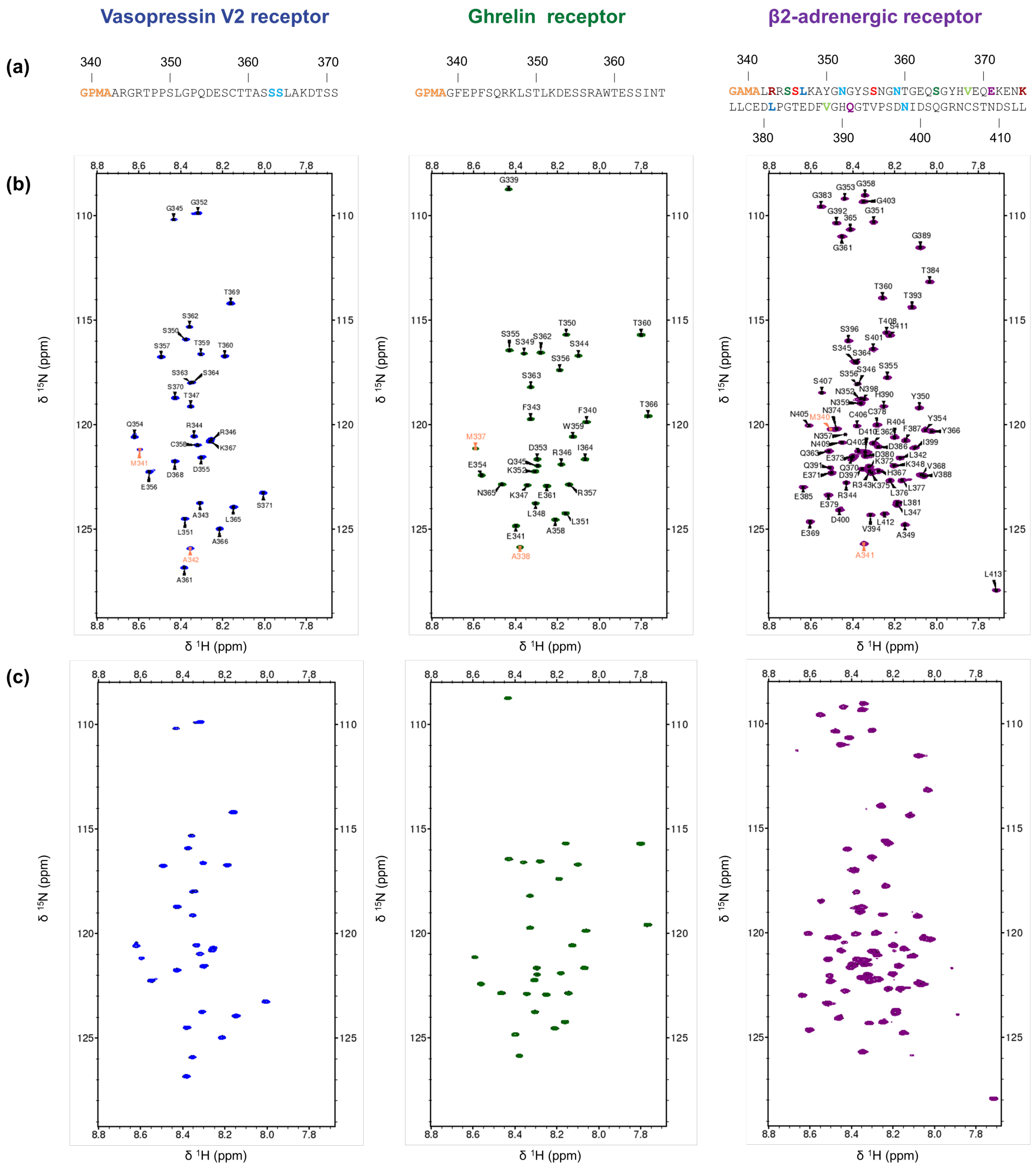


**Figure S3: Related to Figure 3. Assignments of V2R-Cter (blue, left), GHSR-Cter (green, middle) and β2AR-Cter (purple, right). (a)** Sequence of the GPCRs C-termini. Assigned residues are indicated in black while assignments of overlapping cross-peak are noticed in color. Amino acids coming from the protease cleavage site are shown in orange and set of overlapping residues in $^{15}$N-HSQC are in the same color. **(b)** $^{15}$N-HSQCs were recorded on 300 µM samples at 700 MHz, 20 °C, in 50 mM Bis-Tris pH 6.7, 150 mM NaCl buffer. Residue-specific assignments are shown on spectra. Amino acids coming from the protease cleavage site are shown in orange. **(c)** The concentration of GPCR-Cter does not affect their conformations. Overlay of $^{15}$N-HSQCs of V2R-Cter (blue, left), GHSR-Cter (green, middle) and β2AR-Cter (purple, right) recorded on samples at 300 µM (black) or 100 µM (colored) at 700 MHz, 20 °C, in 50 mM Bis-Tris pH 6.7, 150 mM NaCl bufer. No chemical shift changes were observed in NMR spectra at different concentrations and thus, the spectrum at 300 µM (black) is hidden by the spectrum at 100 µM (colored).

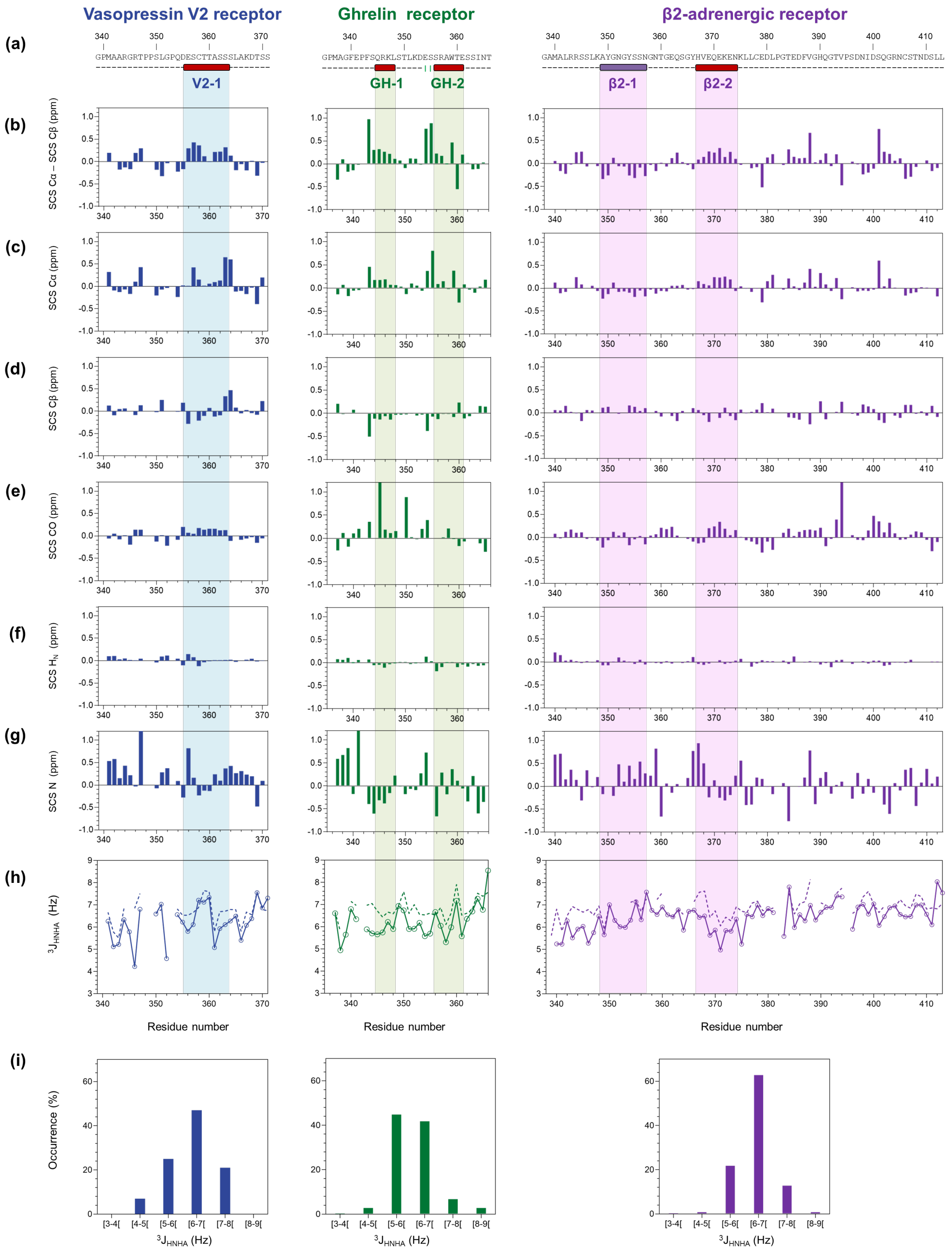


**Figure S4: Related to Figure 3. Secondary chemical shift (SCS) and $^{3}$JHNHA scalar coupling measured for for V2R-Cter (blue, left), GHSR-Cter (green, middle) and β2AR-Cter (purple, right). (a)** Sequence and NMR secondary structure consensus are indicated according to Figure 3. **(b-g)** Secondary chemical shift (SCS) were calculated using random coil chemical shifts database from POTENCI. **(h)** $^{3}J_{HNHA}$ scalar couplings (colored line) along the sequence are compared to predicted couplings (RC_3JHNHa server) for a fully disorder chain (dash colored line). **(i)** Occurrences of $^{3}J_{HNHA}$ for each Cters. The couplings are consistent with an overall unfolded protein (values from 6 to 8 Hz) with a low content of helical and extended conformation (values below 6 Hz and above 8 Hz respectively). SCS were extracted from 3D experiments recorded at 700 MHz (800 MHz for β2AR-Cter), 20 °C, in 50 mM Bis-Tris pH 6.7, 150 mM NaCl buffer and $^{3}J_{HNHA}$ were recorded at 700 MHz, 20 °C, in 50 mM Bis-Tris pH 6.7, 150 mM NaCl buffer.

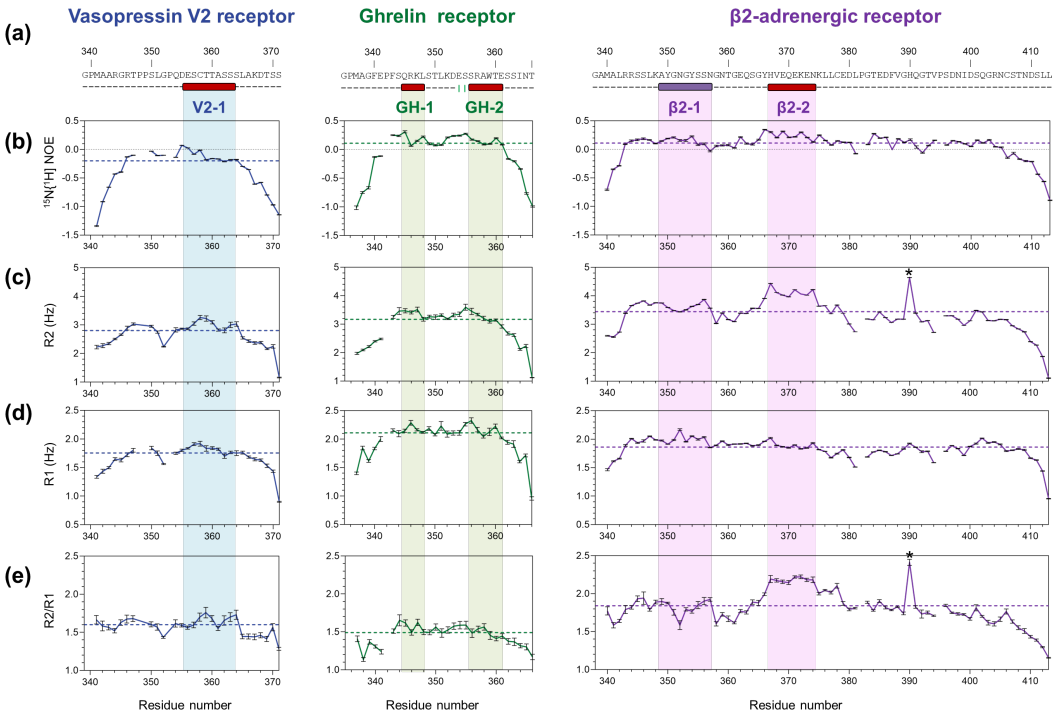


**Figure S5: Related to Figure 3e. Relaxation data of V2R-Cter (blue, left), GHSR-Cter (green, middle) and β2AR-Cter (purple, right). (a)** Sequence and NMR secondary structure consensus are indicated according to Figure 3. **(b)** $^{15}N\{^{1}H\}$ heteonuclear NOE, **(c)** transversal relaxation rates R2, **(d)** longitudinal relaxation rates R1 and **(e)** R2/R1 ratio are shown in colored lines. Average values are indicted by dashed colored line. Stars indicate histidine 390 outlier due its extreme pH sensitivity. Relaxation data were recorded at 700 MHz, 20 °C, in 50 mM Bis-Tris pH 6.7, 150 mM NaCl buffer.

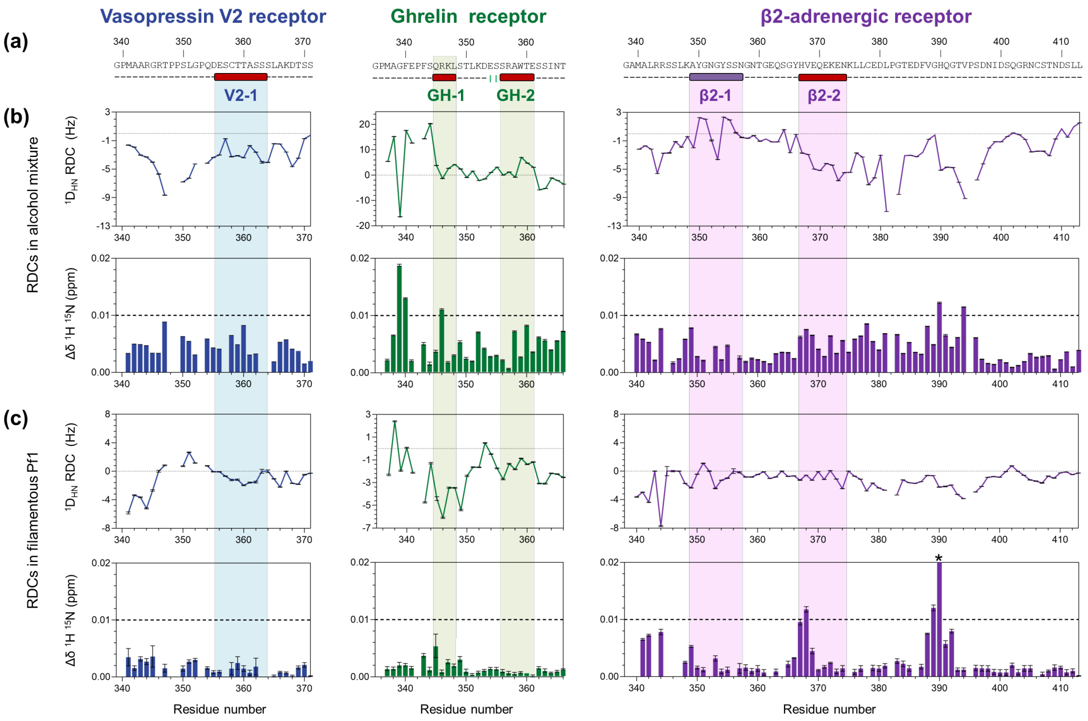


**Figure S6: Related to Figure 3f. $^1D_{NH}$ RDCs values of V2R-Cter (blue, left), GHSR-Cter (green, middle) and β2AR-Cter (purple, right). (a)** Sequence and NMR secondary structure consensus are indicated according to Figure 3. RDCs data measured in **(b)** alcohol mixture or in **(c)** filamentous phage Pf1. In each media, $^1D_{NH}$ RDCs profile (top), chemical shift perturbations (bottom) are shown. Stars indicate histidine 390 outlier due its extreme pH sensitivity. RDCs were measured at 700 MHz, 20 °C, in 50 mM Bis-Tris pH 6.7, 150 mM NaCl buffer.

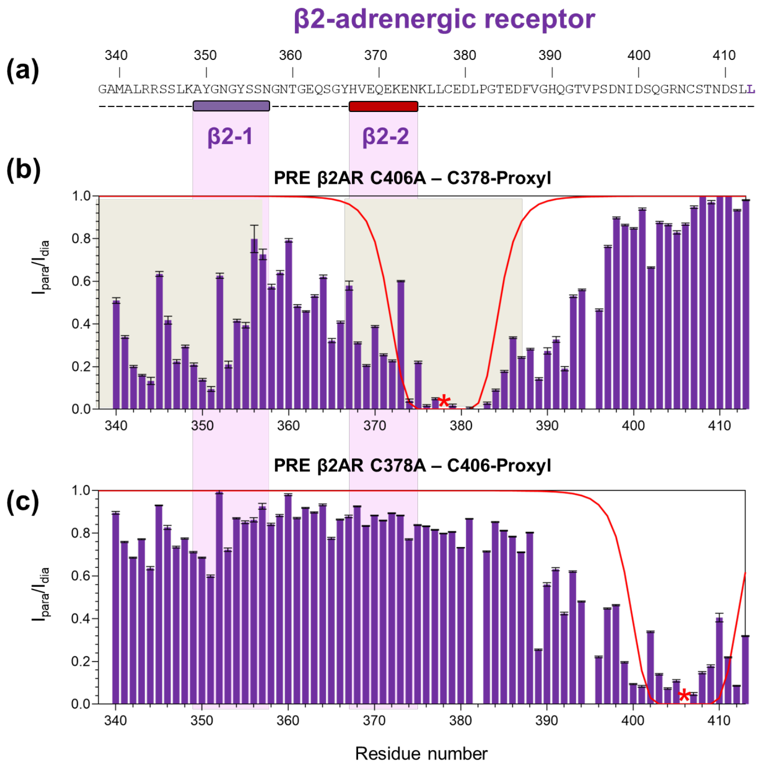


**Figure S7: Related to Figure 3. PRE data of β2AR-Cter variants**. **(a)** Sequence and NMR secondary structure consensus are indicated according to Figure 3. **(b)** PRE data of C406A mutant labeled on C378 (red star). The C378 labeled with the paramagnetic probe, which just lies after β2-2 region, induced a reduction of the intensity ratio in β2-1 region, suggesting long-range interaction. PRE affected regions are highlighted in grey. (c) PRE data of C378A mutant labeled on C406 (red star). The C406 labeled with the paramagnetic probe, does not lead to reduction of intensity ratio. This region does not form long-range interaction with other regions. PREs were calculated from the ratio of intensity ($I_{para}/I_{dia}$) of $^{15}$N-HSQC spectra measured for the paramagnetic ($I_{para}$) sample and for the diamagnetic sample ($I_{dia}$). PRE were recorded at 700 MHz, 20 °C, in 50 mM Bis-Tris pH 6.7, 150 mM NaCl buffer on samples at 50 μM. Theoretical PRE curves for a completely disordered protein are shown in red.

## REFERENCES


1. Uversky, V.N.; Gillespie, J.R.; Fink, A.L. Why Are “Natively Unfolded” Proteins Unstructured under Physiologic Conditions? *Proteins* **2000**, *41*, 415–427, doi:10.1002/1097-0134(20001115)41:3<415::aid-prot130>3.0.co;2-7.
2. Sibille, N.; Bernadó, P. Structural Characterization of Intrinsically Disordered Proteins by the Combined Use of NMR and SAXS. *Biochem Soc Trans* **2012**, *40*, 955–962, doi:10.1042/BST20120149.
3. Cordeiro, T.N.; Herranz-Trillo, F.; Urbanek, A.; Estaña, A.; Cortés, J.; Sibille, N.; Bernadó, P. Small-Angle Scattering Studies of Intrinsically Disordered Proteins and Their Complexes. *Curr Opin Struct Biol* **2017**, *42*, 15–23, doi:10.1016/j.sbi.2016.10.011.
4. Cordeiro, T.N.; Herranz-Trillo, F.; Urbanek, A.; Estaña, A.; Cortés, J.; Sibille, N.; Bernadó, P. Structural Characterization of Highly Flexible Proteins by Small-Angle Scattering. *Adv Exp Med Biol* **2017**, *1009*, 107–129, doi:10.1007/978-981-10-6038-0_7.
